# Propofol‐induced downregulation of NR2B membrane translocation in hippocampus and spatial memory deficits of neonatal mice

**DOI:** 10.1002/brb3.734

**Published:** 2017-06-02

**Authors:** Yuzhu Wang, Song Han, Ruquan Han, Yue Su, Junfa Li

**Affiliations:** ^1^ Department of Anesthesiology Beijing Shijitan Hospital Capital Medical University Beijing China; ^2^ Department of Neurobiology and Center of Stroke Beijing Institute for Brain Disorders Capital Medical University Beijing China; ^3^ Department of Anesthesiology Beijing Tiantan Hospital Capital Medical University Beijing China

**Keywords:** membrane translocation, neonate, NR2B, phosphorylation, propofol, spatial memory

## Abstract

**Background:**

Thousands of infants and children are undergoing anesthesia around the world every day. But impacts of anesthetics on the developing neural system remain unclear yet. Previous evidence showed that anesthesia might affect the developing neural system. Thus, early‐life anesthesia becomes a critical issue in clinical pediatric practice. Hence, propofol, a short‐acting and widely applied intravenous anesthetic, has been gaining focus upon neonatal anesthesia.

**Methods:**

Fifty‐four male C57BL/6J mice were randomly divided into following three groups: group D6 intraperitoneally (i.p.) injected propofol (100 mg/kg body weight) once a day from postnatal day 6 (P6) to P11, group D1 administrated propofol (100 mg/kg, i.p.) at P6 solely and administrated normal saline (10 ml/kg, i.p.) from P7 to P11, and group *N* treated with normal saline (10 ml/kg, i.p.) from P6 to P11 as the control (*n* = 18 per group). Then, at P28, nine mice were collected randomly from each group for NR2B membrane translocation and phosphorylation analysis, and the rest half in each group were assigned to perform Morris water maze tests from P28 to P35.

**Results:**

Results showed that total protein expression levels of NR2B increased (*p* < .001) while its membrane translocation decreased (*p* < .001, *n* = 9 per group) in the hippocampus but not in the prefrontal cortex of neonatal mice after repeated propofol administration. Phosphorylation levels of NR2B at serine 1303 (D1: *p* < .05; D6: *p* < .001, *n* = 9 per group) and serine 1480 (D1: *p* < .01, D6: *p* < .001, *n* = 9 per group) increased significantly as well in the hippocampus compared with group *N*. In addition, memory deficits (*p* < .05, *n* = 9 per group) were observed in Morris water maze tests of group D6 mice.

**Conclusions:**

These results suggested that propofol exposure downregulates NR2B membrane translocation and causes spatial memory deficits, with a mediated increased NR2B protein expression and phosphorylation at Ser1303/1480 residues in the hippocampus of neonatal mice.

**What is already known**

Propofol, a commonly used intravenous anesthetic, blockades NMDA receptors and might affect the developing central nervous system of neonatal mice.

**What this article adds**

Neonate propofol exposure increased NR2B phosphorylation at Ser1303/1480 residues and downregulated NR2B membrane translocation in the hippocampus, accompanying with spatial memory deficits of puberty mice.

## 1 INTRODUCTION

General anesthesia induces temporarily unconscious via proper inhibition of the nervous system. It has been commonly used in clinical pediatrics. But the developing nervous system of infants and young children has potential vulnerability. A transient disruption of synaptic transmission between neurons during early postnatal period may result in reduced cortical changes persisting to puberty and adulthood (Greenhill et al., 2015). Recent randomized trial demonstrated that children's visual and verbal memory impaired 24‐hr after day‐case propofol general anesthesia (Millar et al., 2014), and a cohort study showed an increased risk of neuropsychiatric disorder years after early‐life general anesthesia (Sprung et al., 2012). Given the Food and Drug Administration (FDA) Warning of anesthesia in developing brains in February 2017 (Andropoulos & Greene, 2017), the effect of anesthetic on the developing nervous system is taking into more serious account.

Propofol, a short‐acting intravenous anesthetic, is increasingly used in starting and maintenance of pediatric anesthesia. Propofol acts on γ‐aminobutyric acid‐A receptor and results in widespread inhibition of *N*‐methyl‐d‐aspartate (NMDA) receptor (Irifune et al., 2003; Lingamaneni, Birch, & Hemmings, 2001). NMDA receptor blocking has been proven to be associated with narcotism via genetic approaches (Sato, Kobayashi, Murayama, Mishina, & Seo, 2005). Blockade of NMDA receptor causes neuron apoptotic in the cerebral cortex of neonatal mice (Kaushal, Tamer, Opoku, & Forcelli, 2016).

As a kind of excitatory glutamate ion channel, NMDA receptor is a heterotetramer or heteropentamer consisting of NR1, NR2 (NR2A‐D), or NR3 (NR3A‐B) subunit (Monaco, Gulchina, & Gao, 2015). NR1 homodimer is an obligatory subunit of NMDA receptor while NR2B is related with learning and memory. NR2B subunit is more dynamic than other NMDA subunits (Bharadwaj et al., 2014; Lussier, Sanz‐Clemente, & Roche, 2015; Plattner et al., 2014). It has been demonstrated that NR2B tends to be recycled to the plasma membrane after internalization, whereas other NR2 subunits are mainly sorted to degradation (Lussier et al., 2015). Actually, NMDA receptor has a highly refined control of long‐term potentiation (LTP) and long‐term depression (LTD), which directly and specifically implicated in learning and memory (Sweatt, 2016). Changes in LTP and LTD of synaptic strength are regulated by NR2B‐modified NMDA receptor transport (Montgomery, Selcher, Hanson, & Madison, 2005; Tang et al., 2015).

Meanwhile, NR2B transport closely relates to NR2B phosphorylation (Lussier et al., 2015). Although there are many phosphorylation sites on C‐terminal of NR2B, it was noticed that phosphorylation of NR2B Ser1303 acts as an initial step of NMDA‐induced excitotoxicity (Li et al., 2016), while phosphorylation of NR2B Ser1480 may contribute to neuroprotection of cortical neurons during NMDA receptor activation (Cook et al., 2011). Hence, it is meaningful to focus on this two potential treatment targets. Notably, phosphorylation of Ser1303 and Ser1480 (Ser1303/1480) particularly interacts with CaMKII‐involved kinase pathways in regulation of NMDA receptor transport (Prabhu, Suma, Mayadevi, & Omkumar, 2012; Sanz‐Clemente, Gray, Ogilvie, Nicoll, & Roche, 2013; Sanz‐Clemente, Matta, Isaac, & Roche, 2010), while CaMKII phosphorylation was reported to be involved in propofol‐induced narcotization and propofol‐related spatial memory deficit (Cui et al., 2009; Gao, Peng, Xiang, Huang, & Chen, 2014). Thus, we hypothesized that propofol might affect NR2B transport with changed NR2B phosphorylation level at Ser1303/1480 and might affect hippocampal‐related spatial memory in the developing nervous system.

Importantly, the impact of propofol on the central nervous system depends on its developmental stage (Briner et al., 2011). It has been demonstrated that between postnatal day 5 (P5) and P10, both length and number of apical and basal dendritic increase dramatically in rat medial prefrontal cortex (Briner et al., 2011). More, in the nervous system, density of NR2B sudden droops down around P7, since which time the predominance of NR2 subunit starts to switch and new generated synapses lack of NR2B (Liu, Murray, & Jones, 2004). Thus, considering the critical early postnatal period and the feasibility of experimental approach, we set P6 as the first day of propofol exposure. Besides, since updating of spatial memory has a co‐hippocampal‐prefrontal direct pathway during memory afferentiation (Spellman et al., 2015), we took the prefrontal cortex as well to exclude its potential responsibility of spatial memory changes. Hence, we combined protein analysis of NR2B expression with the Morris water maze test to reveal the effect of early‐life propofol exposure and its influence between NR2B transport and NR2B Ser1303/1480 phosphorylation.

## 2 MATERIALS AND METHODS

### 2.1 Animal and treatments

The Animal Experiments and Experimental Animal Welfare Committee of Capital Medical University approved this study (ACUC code: AEEI‐2015‐031). The C57BL/6 mice were raised in laboratory animal room at specific pathogen–free (SPF) grade. Mice were fed food and water ad arbitrium in 12‐hr light/dark circulation. Room temperature was maintained at 20–23°C with relative humidity around 40%–70%. At P6, 54 male littermates were divided randomly into three groups (*n* = 18 per group) as follows: The mice in group D6 were administered intraperitoneally (i.p.) with propofol (100 mg/kg body weight, 10 mg/ml concentration, Fresenius Kabi, J20080023, Bad Homburg, Taunus, Germany) daily from P6 to P11; group D1 mice were administered with propofol (100 mg/kg, i.p.) once at P6 and then treated with same volume of normal saline (10 ml/kg, i.p.) from P7 to P11; and group *N*, as the control, were injected with normal saline (10 ml/kg, i.p.) from P6 to P11. The exposure dose of propofol was made conversion according to body surface area between human and mice. And the calculation dose was based on the clinical package insert of propofol which could induce surgical anesthesia in human children. Respiratory rates were observed continuously during propofol anesthesia.

Respiratory rates of neonatal mice were 139.7 ± 6.1 counts per min (*n* = 27) before anesthesia in our observation. Thus, when respiratory rates were lower than 110.0 counts per min (20% declined), breath was stimulated via tapping neonatal planta pedis or tails with torso massaging. Because neonatal mice have inadequate physical strength to roll over, righting reflex was defined as body twisting or limbs swing at abdomen‐up position. Righting reflex recover was regarded as emergence from anesthesia. Forty min after propofol or normal saline administration, blood gas analysis (OPTI Medical Systems Inc, GA, USA) was performed in P6 mice. Intralipid was not used in this study as a separate control according to report that there is no different impact in nervous system between intralipid and normal saline via peritoneal injection (Yang et al., 2014). During propofol anesthesia, pups were separated from the maternal temporarily and kept in warming blanket to maintain normal body temperature. After the 6‐day treatment, all pups were feeding with their maternal side until ablactation at P21. Body weights were measured once a day since P6.

### 2.2 Western blot analysis

Mice were sacrificed at P28. Then, the prefrontal cortex and hippocampus were isolated at 4°C immediately and stored at −80°C until use. Tissue were homogenized with buffer A [50 mM Tris‐Cl, 5 mM sodium pyrophosphate, 100 μM sodium vanadate, 2 mM ethylenediaminetetraacetic acid, 2 mM ethylenebis(oxyethylenenitrilo)tetraacetic acid, 1 mM dl‐dithiothreitol, potassium fluoride protease inhibitor, and phosphatase inhibitor (#4038377 024523 and #4038377 024127, Roche, Basel, Switzerland)] on ice. The homogenate was centrifuged (30,000 *g*, 30 min) at 4°C and the supernatant were reserved as cytosol. The pellet was completely redissolved in buffer C (buffer A with 2% SDS) and sonicated as particulate fraction before quantitation of protein concentration via BCA protein assay reagent (Thermo Fisher Scientific, MA, USA). The SDS‐polyacrylamide gel electrophoresis (PAGE) was used to transfer proteins onto a polyvinylidene fluoride (PVDF) membrane (GE Healthcare Life Sciences, UK) using electrophoresis.

The antibodies of anti‐p‐Ser1303 NR2B (1:500 dilution, #ab81271, Abcam, Cambridgeshire, UK), anti‐p‐Ser1480 NR2B (1:1,000 dilution, #NB100‐61103, Novus Biologicals, CO, USA), anti‐NR2B antibody (1:250 dilution, #sc‐9057, Santa Cruz, CA, USA), and anti‐β actin (1:10,000 dilution, #60008‐1‐Ig, Proteintech, IL, USA) were incubated overnight at 4°C, respectively. After the incubation of secondary antibodies that horseradish peroxidase‐conjugated goat anti‐rabbit IgG or goat anti‐mouse IgG (1:400 dilution, #111‐035‐003 or #115‐035‐003, Jackson, PA, USA), the chemiluminescent signals (ECL kit, Thermo Fisher Scientific, Waltham, MA, USA) were captured and analyzed using the gel imaging and analysis system (Thermo Fisher Scientific, Waltham, MA, USA).

### 2.3 Morris water maze test

Researcher who performed Morris water maze tests remained blinded to the grouping throughout the study period. Morris water maze tests were performed from P28 to P35. A 120‐cm‐diameter circular pool (Coulbourn Instruments, PA 18052, USA) with white‐dyed water maintained between 20 and 23°C was enclosed with four different extra‐maze cues (Figure 3c) fixed at four quartiles of the pool periphery. A 10‐cm‐diameter hyaline platform was hidden 1 cm beneath opaque water surface in the second quadrant of the pool (Figure 3c II quadrant). During the acquisition phase, one block with four trials was carried out per day from four different quadrant of the pool for five consecutive days since P28. Every trial was started since lowering mice into water and terminated until the mice found the platform or 90 s elapsed, during which the escape latency and swim path were recorded. Then, the mouse was let on the platform for 20 s before the next trial. While at the sixth day for probe trial, the same 4‐trial was conducted 90 s without the platform, quadrant time (%) and average speed (cm/s) were recorded. At the seventh and eighth day, the platform was replaced with a visible black cylinder landmark above the water surface while the four extra‐maze cues were removed; escape latency and swim path recorded ditto.

### 2.4 Statistical analysis

Using Quantity One (version 4.6.2, Gel Doc 2000 imaging system, Bio‐Rad Company, Hercules, CA 94547, USA) software to perform the quantitative analysis of western blot, the band densities of NR2B and its phosphorylated form were firstly rectified by β‐actin, and then normalized to 100% for each set experiment. The protein expression (band densities both in cytosol and particulate for each specimen), membrane translocation (band density in particulate/band densities both in cytosol and particulate for each specimen), and phosphorylation at Ser1303/1480 (band densities of phosphorylated form both in cytosol and particulate for each specimen) levels of NR2B were calculated as 100% in *N* group, and then D1 and D6 groups were expressed as percentage of that of *N* group. All data were analyzed using an one‐way ANOVA and Bonferroni test for multiple comparisons (SPSS software version 21). A *p*‐value <.05 was considered statistically significant, and all ultimately presentations were mean ± *SD*.

## 3 RESULTS

**Figure 1:**
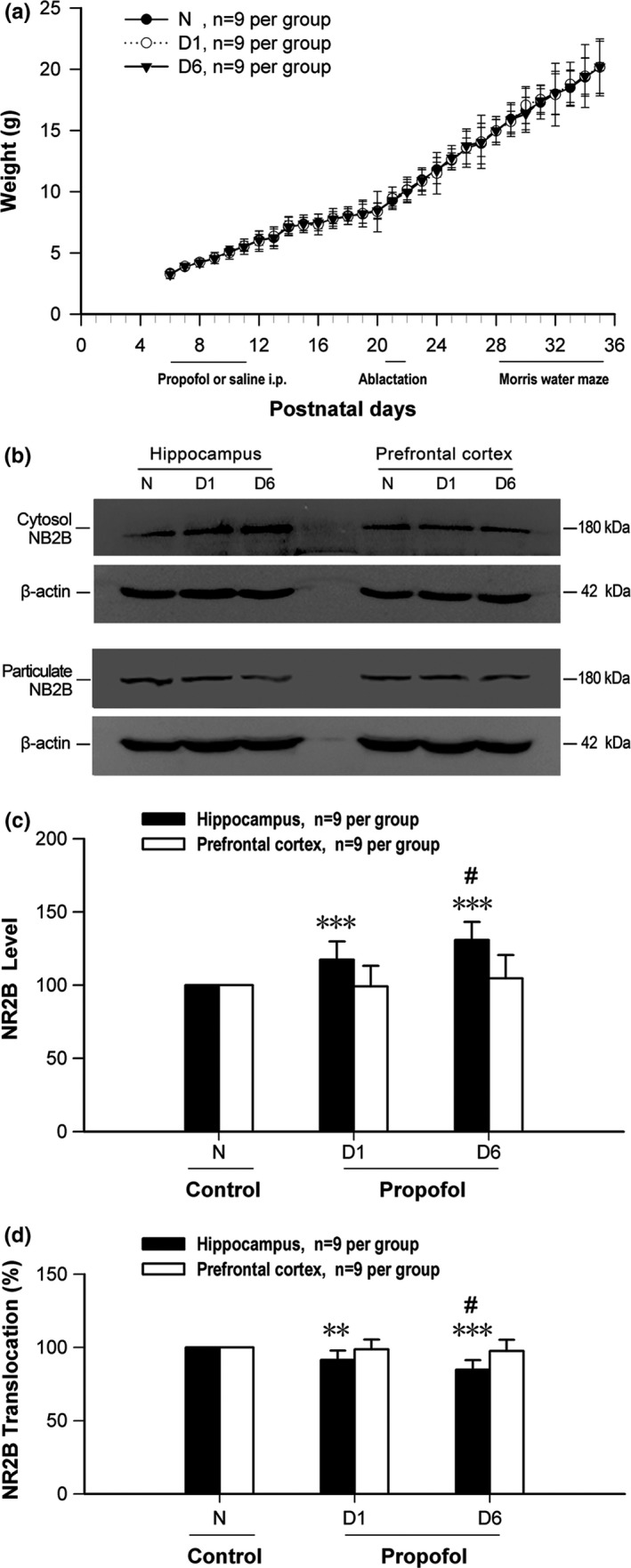
Changes in NR2B protein expression and membrane translocation in the hippocampus and prefrontal cortex of P28 mice after early‐life propofol exposure. (a) Changes in body weight and experimental schedule from P6 to P35. (b) Typical results of western blot showed the changes in NR2B protein levels in cytosol and particulate fractions of the hippocampus and prefrontal cortex of P28 mice after early‐life propofol exposure. The quantitative analysis results demonstrated that the total protein expression levels of NR2B increased (c), while the NR2B membrane translocation decreased significantly (d) in the hippocampus but not in the prefrontal cortex of neonatal mice with repeated propofol administration. **p < .01, ***p < .001 versus control *N* group; ^#^ *p* < .05 versus corresponding D1 group; *N*, normal saline (10 ml/kg); D1, propofol (100 mg/kg) solely at P6; D6, propofol (100 mg/kg) P6 to P11; *n* = 9 per group

No significant difference was observed in body weight (Figure 1a) among groups. Blood gas data were also undifferentiated at 40 min after propofol and normal saline administration of P6 mice (pH: 7.45 ± 0.05 vs. 7.41 ± 0.05, *p* = .20; PCO_2_: 43.01 ± 4.28 mmHg vs. 45.18 ± 4.18 mmHg, *p* = .29; PO_2_: 82.93 ± 10.06 mmHg vs. 82.83 ± 5.74 mmHg, *p* = .97, *n* = 9 per group). Average time of anesthesia emergence at P6 is 73.22 ± 3.75 min (*n* = 9 per group).

### 3.1 Effects of propofol on NR2B protein expression and membrane translocation in the hippocampus and prefrontal cortex of neonatal mice

The typical results of western blot in Figure 1b showed that, following early‐life propofol exposures, protein levels of NR2B were increased in hippocampal cytosol but change little in cytosol of the prefrontal cortex. No changes of NR2B protein levels were detected in particulate, neither in the hippocampus nor in the prefrontal cortex of neonatal mice after early‐life propofol exposures. Total protein levels of NR2B increased significantly in hippocampus (*p* < .001) but not in prefrontal cortex (*p* > .05) of neonatal mice after propofol administration (Figure 1c, n = 9 per group). However, NR2B membrane translocation decreased in hippocampus (*p* < .001) but not in prefrontal cortex (*p* > .05) of neonatal mice with repeated propofol administration (Figure 1d, n = 9 per group). These results suggested that propofol exposure could cause the increased protein expression and subcellular redistribution of NR2B in the hippocampus, while NR2B in the prefrontal cortex was less vulnerable of neonatal mice.

### 3.2 Effects of propofol exposure on NR2B phosphorylation level in the hippocampus and prefrontal cortex of neonatal mice

**Figure 2:**
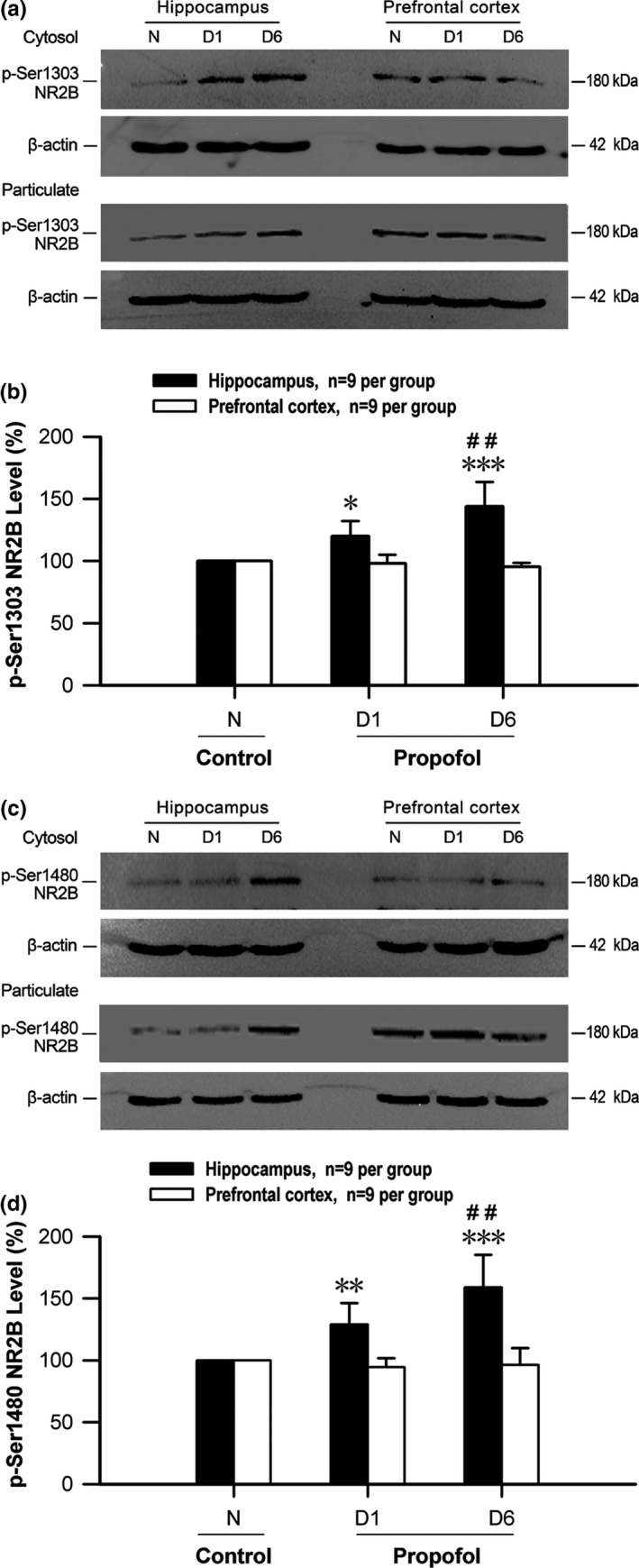
Changes of NR2B phosphorylation levels in the hippocampus and prefrontal cortex of P28 mice after early‐life propofol exposure. Typical results of western blot showed the changes in p‐Ser1303 (a) and p‐Ser1480 (c) NR2B phosphorylation levels in cytosol and particulate fractions of the hippocampus and prefrontal cortex of P28 mice after early‐life propofol exposure. Quantitative analysis results demonstrated that the phosphorylation levels of p‐Ser1303 NR2B (b) and p‐Ser1480 NR2B (d) increased significantly in the hippocampus but not in the prefrontal cortex of P28 mice after early‐life propofol exposure. *p < .05, **p < .01, ***p < .001 versus control *N* group; ^##^ *p* < .01 versus corresponding D1 group, *n* = 9 per group

To explore the possible regulatory mechanism of NR2B membrane translocation, the effects of early‐life propofol exposure on NR2B phosphorylation levels were further observed in the hippocampus and prefrontal cortex of neonatal mice. We found that the phosphorylation levels of NR2B at serine 1303 (p‐Ser1303) increased significantly (D1: *p* < .05; D6: *p* < .001) in the hippocampus of neonatal mice following propofol exposure (Figure 2a,b, *n* = 9 per group) when compared with that of normal control *N* group. Similarly, the early‐life propofol exposure also caused the significant increase in NR2B phosphorylation levels at serine 1480 (p‐Ser1480) in the hippocampus (not prefrontal cortex) of neonatal mice of D1 and D6 groups (Figure 2c,d, D1: *p* < .01, D6: *p* < .001, *n* = 9 per group). Consistent with previous reports that the transport of NMDA receptors and NR2B synaptic ratio are regulated by phosphorylated modification and synaptic activity (Lussier et al., 2015; Sanz‐Clemente et al., 2010;), these results indicated that the increase in p‐Ser1480 and p‐Ser1303 levels of NR2B might be responsible for the decreased membrane translocation in the hippocampus of neonatal mice after propofol exposure.

### 3.3 Propofol‐treated neonatal mice performed spatial memory deficits during adolescent period

**Figure 3:**
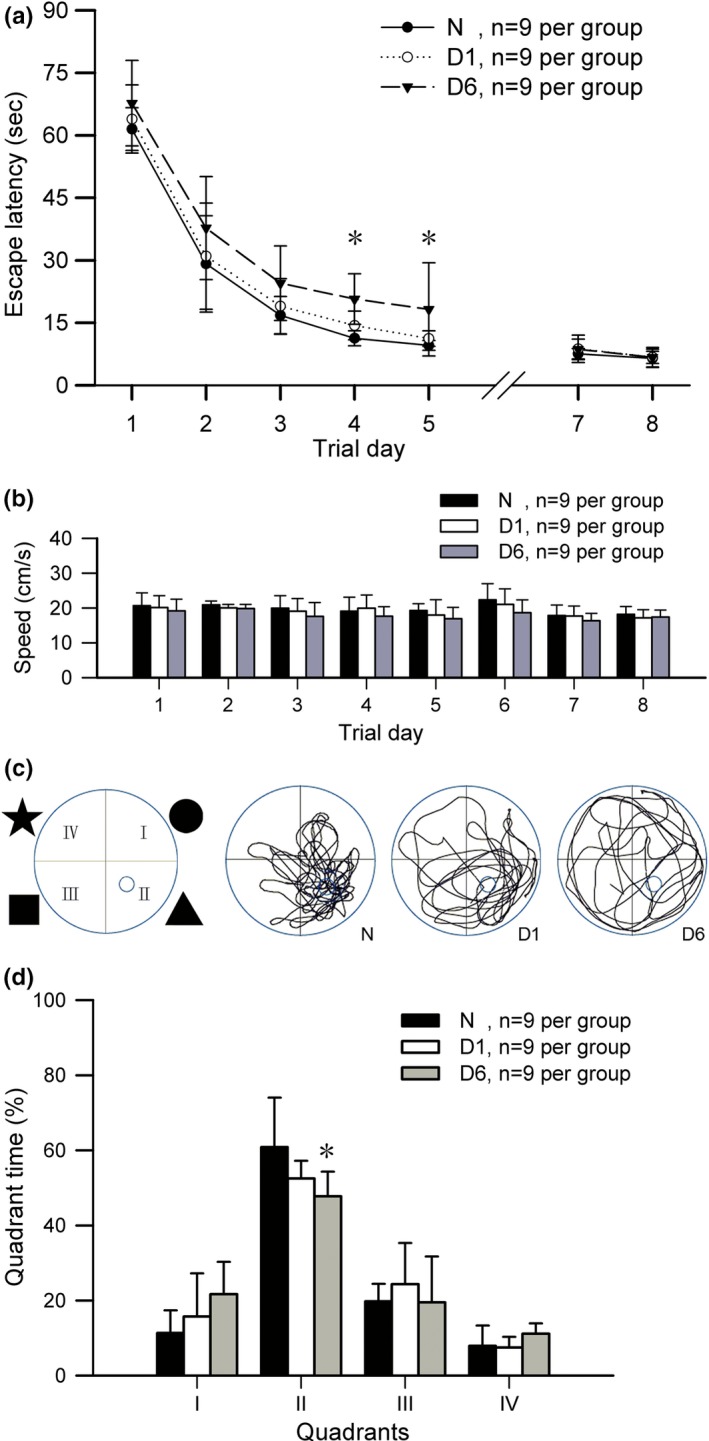
Effects of propofol exposure on spatial memory of neonatal mice. (a) Escape latency results showed that early‐life propofol exposure could significantly affect the spatial memory process of locating fixed hidden but not the visual platform in neonatal mice. (b) The results of average speeds showed that there were no statistical significances in motoric strength among groups from trial day 1 to 8. (c) Indicating the four extra‐maze cues and the typical swimming path of mice from *N*, D1 and D6 groups. (d) The trace analysis results showed that the mice spent less swimming time in the second quadrant (hidden platform location) at sixth day after early‐life propofol exposure. *p < .05 versus control *N* group, *n* = 9 per group

As propofol exposure could cause the alterations in subcellular distribution and phosphorylation at Ser1303 and Ser1480 of NR2B in the hippocampus, the spatial memory was further observed using the Morris water maze in D1 and D6 mice from P28 to P35. The initial five trial days were defined as acquisition phase while the sixth day as probe trial. The escape latency during acquisition phase revealed a learning process of locating fixed hidden platform. As shown in Figure 3a, mice in group D6 spent more time to locate the hidden platform, and the significant delay could be found at trail day 4 and 5 (*p* < .05, *n* = 9 per group) when compared with that of the control *N* group. In addition, the mice from group D6 showed a significant deficit in memory for the hidden platform (quadrant II) in probe trial of the sixth trail day when compared with that of group *N* (*p* < .05, *n* = 9 per group, Figure 3c,d). No significant difference was observed between group *N* and D1 during acquisition phase and the sixth trail day (*p* > .05, *n* = 9 per group, Figure 3a,d). As both the vision and motoric strength of mice could affect the reliability of Morris water maze tests, the escape latency was redetermined using a visible platform at the same location of quadrant II at the seventh and eighth trail day. As shown in Figure 3a,b, no significant differences in escape latency and average speed were observed among groups (*n* = 9 per group). These results suggested that propofol exposure could induce spatial memory deficits of neonatal mice without affecting their visual and moving abilities.

## 4 DISCUSSION

Our study showed downregulated NR2B transport accompanied by spatial memory deficits after early‐life propofol exposure. Analogously, previous researches demonstrated that genetically reducing NR2B transport result in mice memory deficits (Plattner et al., 2014; Takei, Kikkawa, Atapour, Hensch, & Hirokawa, 2015). It has been known that NMDA receptors act a critical part in spatial memory encoding (Farina & Commins, 2016), and recent study showed that NR2B may be more important than other NMDA receptors during hippocampal LTP induction and spatial memory performance (Zhang, Xing, Wang, Tao, & Cheng, 2015). Thus, the spatial memory deficits observed in this study might probably associate with downregulated NR2B membrane translocation after early‐life propofol exposure. Yet, further electrophysiological studies will continue to build direct evidences about connections between NR2B membrane translocation and spatial memory deficits.

We also found that NR2B phosphorylation increased with downregulated NR2B membrane translocation in the hippocampus after early‐life propofol exposure. Although previous studies has demonstrated that phosphorylation is a key mechanism of NR2B trafficking regulation (Lussier et al., 2015), it was insufficient, in our study, to support the relationship between changes of propofol‐induced NR2B phosphorylation and propofol‐induced membrane translocation, which we will go further exploration. Nevertheless, previous studies has demonstrated that phosphorylation of Ser1480 decreases NR2B membrane translocation (Chung, Huang, Lau, & Huganir, 2004; Sanz‐Clemente et al., 2010), and we observed similar phenomenon after early‐life propofol exposure in this study. Genetic manipulation has proven that regulation of NR2B membrane translocation via phosphorylation is important for learning and memory (Maier et al., 2013; Yin, Feng, Takei, & Hirokawa, 2012). Given the increased phosphorylation levels of Ser1303/1480 and decreased NR2B membrane translocation with spatial memory deficits we found, there might be a hypothesis that early‐life propofol exposure result in puberty memory deficits via phosphorylation‐based regulation of NR2B membrane translocation.

Besides, we observed that propofol increased both p‐Ser1303 and p‐Ser1480 levels. Although it has been elaborated that those two serine phosphorylation sites share some common kinases and signaling pathways in previous researches (Prabhu et al., 2012; Sanz‐Clemente et al., 2013; Yan et al., 2011), an original study indicated that Ser1480 phosphorylation does not affect p‐Ser1303 level (Sanz‐Clemente et al., 2013). Therefore, our preliminary study needs further integration of kinases‐involved pathways of how propofol affects these two phosphorylation sites, and in the next study, genetic approaches and mutational pragmatic data can improve this.

It is interesting that propofol exposure at P6‐P11 would have continued biochemical effects at P28. Since propofol has been metabolized by P28, there might be some other consequences of early‐life propofol exposure that causes these later biochemical changes. Phosphorylation of proteins typically occurs very quickly and is degraded very quickly. So, there likely be an ongoing pathology after propofol is no longer present. It was demonstrated in previous studies that propofol suppresses neurogenesis (Huang et al., 2015) and induces cell apoptosis in developing hippocampus (Sun, Liang, & Pei, 2015). Additionally, propofol was also reported to inhibit expression of embryo myelin basic protein (Guo et al., 2015), which is crucial to axons myelination and nerve conduction in the nervous system. Moreover, it was demonstrated that propofol could induce early‐life morphologic modifications of dendritic spine (Briner et al., 2011), and those morphologic changes persist to adolescent and adult period (Briner et al., 2011). Yet for all this, none did them notice the biochemical changes of NR2B membrane translocation and phosphorylation after early‐life propofol exposure. So, the mechanism of these propofol‐caused later changes is our further focus.

We also noticed that NR2B changes differently between the hippocampus and prefrontal cortex after propofol administration. This may due to different performance of those two regions. Since NR2B acts as a subunit of glutamate NMDA receptor, it has been demonstrated that, in the hippocampus, higher functional connectivity is highly associated with higher glutamate levels, while inversely, higher functional connectivity is associated with lower glutamate levels in the prefrontal cortex (Wagner et al., 2016). In our study, NR2B transport and phosphorylation in the prefrontal cortex was unaffected after early‐life propofol exposure, suggesting that propofol exposure alters hippocampal NR2B homeostasis in a region‐specific manner. Thus, propofol had a greater impact on hippocampal‐related spatial memory in neonatal mice.

In this study, we determined NR2B transport and phosphorylation changes before water maze tests (at P28) as previous research (Zhang, Shen, Xu, & Zhao, 2016). Since there are evidences indicated that Morris water maze training could alleviate memory impairments and change protein expression in mice (Martinez‐Coria et al., 2015). Moreover, at the end of ethology tests, mice could have attained sexual maturity. However, it has been known that sex steroidal hormones change density and phosphorylation of NMDA receptor (Vedder, Smith, Flannigan, & McMahon, 2013) and influence learning and memory performance (Jia, Cui, Song, Yan, & Huo, 2015). Although previous research has been reported the undifferentiated impact of the nervous system between intralipid and normal saline via peritoneal injection (Yang et al., 2014), it was yet a defect that we failed to use intralipid as a separate control in this study.

In conclusion, our findings revealed downregulation of NR2B membrane translocation and increased NR2B phosphorylation in the hippocampus with later spatial memory deficits presenting in mice after early‐life propofol exposure. These results indicate that propofol could disturb intracellular homeostatic stabilization of NR2B in the developing nervous system and result in spatial memory deficits of puberty mice.

## DISCLOSURES

The Animal Experiments and Experimental Animal Welfare Committee of Capital Medical University approved this study (ACUC code: AEEI‐2015‐031). This work was supported by The National Natural Science Foundation of China (31471142 and 31671205) and Beijing Natural Science Foundation (7141001). And there is none conflict of interest.
